# Clinico-Radiological Presentation and Management of Gallbladder Paraganglioma: A Systematic Review

**DOI:** 10.7759/cureus.101244

**Published:** 2026-01-10

**Authors:** Fiza Ismail, Arham Amir Khawaja, Syed Haider Hassan, Hafiz Sohail Ahmad, Saad Abdullah Dar, Rabia Rehman, Haysum Khan, Adeena Azam, Nusrat Fatima, Haseeb Mehmood Qadri

**Affiliations:** 1 General Surgery, Lahore General Hospital, Lahore, PAK; 2 Nephrology, Evercare Hospital, Lahore, PAK; 3 General Surgery and Surgical Oncology, Shaikh Zayed Medical Complex, Lahore, PAK; 4 Neurological Surgery, University of Wisconsin, Madison, USA; 5 Neurological Surgery, Punjab Institute of Neurosciences, Lahore, PAK; 6 General Surgery, Aziz Bhatti Shaheed Teaching Hospital, Gujrat, PAK; 7 Obstetrics and Gynecology, Sharif Medical and Dental College, Lahore, PAK; 8 Neurological Surgery, Shifa Tameer-E-Millat University, Shifa College of Medicine, Islamabad, PAK; 9 Internal Medicine, Allama Iqbal Medical College, Lahore, PAK; 10 Respiratory Medicine, Portsmouth Hospitals University NHS Trust, Portsmouth, GBR

**Keywords:** cholecystectomy, extra-adrenal paraganglioma, gallbladder neoplasm, gallbladder paraganglioma, paraganglioma, surgery, treatment outcomes

## Abstract

Gallbladder paraganglioma (GPG) is a rare neuroendocrine tumour discovered incidentally on final histopathological examination. The objective was to evaluate the clinical findings and management strategies using relevant literature for reported cases of gallbladder paraganglioma. A Preferred Reporting Items for Systematic Reviews and Meta-Analysis (PRISMA)-guided literature search was conducted using data from PubMed, Scopus, and Google Scholar to identify the studies with histopathologically confirmed cases of GPG published between January 2000 and December 2024. Study quality was assessed using the Joanna Briggs Institute checklist, and data analysis was done using descriptive statistics and tabulations. A total of 13 case reports were selected, satisfying the inclusion and exclusion criteria. Among 13 reported cases, 10 (76.3%) were females. The mean age of the patients was 53.8 ± 12.59 years. Right hypochondrial pain was the most common presentation in seven (53.9%) cases, while three (23.1%) cases were asymptomatic. Ultrasound was the initial imaging modality in seven (53.9%) patients, followed by computed tomography scans in six (46.2%). Histopathology showed a nested pattern of chief and sustentacular cells in five (38.5%) cases. On immunohistochemistry, 11 (84.6%) cases were positive for synaptophysin A and nine (69.2%) were positive for chromogranin A. Laparoscopic cholecystectomy was the most common procedure performed in 10 (76.3%) cases. The average duration of follow-up was 66.5 ± 82.10 weeks, which was mentioned for only five patients. GPG should be considered in the differential diagnosis of gallbladder and biliary disorders presenting with features of acute cholecystitis and non-functional tumours. Patients often present with non-specific symptoms aligning with mass effects and cholecystitis. When a growth is suspected, it should be thoroughly evaluated before surgery to rule out other types of invasive gallbladder tumours. If investigations confirm a non-metastatic lesion, proceeding with a simple cholecystectomy is appropriate.

## Introduction and background

Pheochromocytoma and paragangliomas (PPGLs) are rare neuroendocrine tumours arising from the chromaffin cells of the sympathetic and parasympathetic ganglia [1]. Their incidence is around 0.6 cases per 100,000 patients/year [2]. According to a study from Holland, the annual incidence of PPGLs was 4.6 and 1.1 cases per million inhabitants, respectively, which had increased compared to previous years, most likely due to improved diagnostic techniques and clinical awareness [1]. The incidence may be underestimated, as autopsy studies have shown that up to 50% of PPGLs were not clinically suspected [3]. Pheochromocytomas (PCCs) arise from the adrenal medulla, while paragangliomas (PGLs) arise from an extra-adrenal origin. Paragangliomas most commonly arise from sympathetic paraganglia (85% below the diaphragm) or from parasympathetic paraganglia in the head and neck and along the vagus nerve anterior and middle mediastinum, while PPGLs can arise in these extra-adrenal locations, involvement of the gallbladder is exceedingly uncommon [3]. The clinical manifestations of PPGLs vary based on their functionality, specifically their ability to secrete catecholamines. Functional PPGLs typically present with a classic triad of symptoms: palpitations, headaches, and excessive sweating, whereas non-functional tumours are characterised by the lack of these classic symptoms and minimal or absent catecholamine secretion [2]. The biochemical investigation for the detection of PPGLs includes measurement of plasma and/or 24-hour urinary concentration of metanephrines (MNs) (high sensitivity of 97% for detecting PPGLs) [4]. The anatomical localisation is done by computed tomography (CT) scan and magnetic resonance imaging (MRI), as the first-line imaging due to their high sensitivity [4]. Nearly 13 genes are associated with germline mutations, which can manifest as a hereditary form of paraganglioma (PGL) [5,6]. 

The treatment of choice for PGLs is surgical removal of the tumour, as most of the PGLs are benign and can be excised completely [6]. However, all patients with a history of PCC/PGL, especially those with features, such as extra-adrenal location, young age <20 years, noradrenergic or dopaminergic biochemical phenotype, high chromogranin A levels, multiplicity, and germline mutations (SDHA/B), remain at high risk of recurrence; therefore, they are advised to be followed for a longer period [3]. However, evidence on gallbladder paraganglioma recurrence is limited, as published data remain scarce.

Gallbladder paragangliomas (GPGs) are extremely rare paragangliomas, and very few cases have been reported in the literature so far. Due to the scarce literature, no standard guidelines exist on the accurate diagnosis and optimal management of GPGs. This systematic review aimed to compile the existing literature on the gallbladder paragangliomas to suggest the algorithm management of this rare entity.

## Review

Methodology

A Preferred Reporting Items for Systematic Reviews and Meta-Analysis (PRISMA) guided systematic review was conducted between January 2000 and December 2024, incorporating all histopathologically proven cases of GPG. This systematic review is registered with PROSPERO with registration ID CRD42023443753. Only case reports were published on the topic, and therefore, we included only case reports. The articles were selected based on the following inclusion/exclusion criteria. Inclusion criteria: It included case reports and case series of histopathologically confirmed gallbladder paragangliomas, studies involving living human subjects only, articles published in English or with a reliable translated version available and publications with complete and accessible full-text content. Exclusion criteria: It included editorials, letters to the editor, commentaries, and conference abstracts without sufficient case details, animal studies and cadaveric studies and duplicate publications or studies with incomplete clinical or histopathological data.

Search Strategy

**Figure 1:**
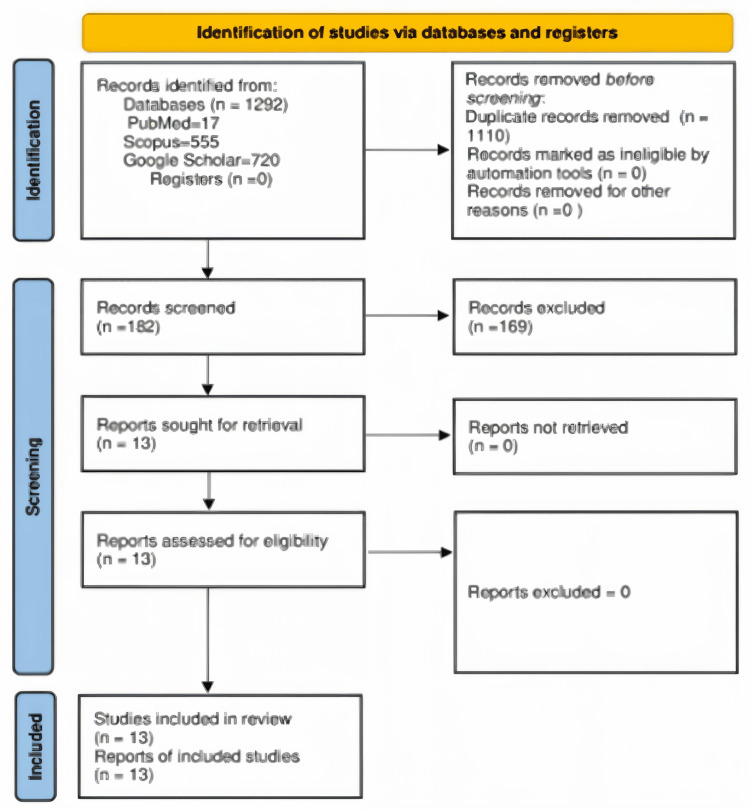
Preferred Reporting Items for Systematic Review and Meta-Analysis (PRISMA) flow chart for search strategy and the quality assessment for included studies. The set exclusion criteria, as detailed above, were the reason for excluding various studies.

The selected case reports and series were collected from databases and search engines, namely, PubMed, Scopus, and Google Scholar (Figure 1). Boolean operators (AND/OR) with keyword combinations were used to collect data from database search engines. The keyword combination is as follows: "Gall Bladder Paraganglioma" OR "Paraganglioma of Gall Bladder" OR "Paraganglioma Gall Bladder" OR "Gallbladder Paraganglioma" OR "Paraganglioma of Gallbladder" OR "Paraganglioma Gallbladder" OR "Gall Bladder" OR Gallbladder AND "Paraganglioma" OR "Extra-adrenal Pheochromocytoma" OR "Neuroendocrine Tumour" OR "Chromaffin Tumour".

Quality Assessment

The quality assessment was done using the eight-component Joanna Briggs Institute (JBI) Critical Appraisal guidelines. The checklist assesses the domain, including clinical history, diagnostic workup, treatment, outcomes achieved, and the clinical significance of the case [7]. The JBI checklist is mentioned in Appendix A.

Data Extraction and Manuscript Writing

After quality assessment by the Joanna Briggs Institute criteria, data were extracted from selected articles by four authors (RR, HK, AA, NF). Included articles were screened manually. The following variables were recorded: demographic details, clinical findings, radiological investigations, biochemical and histopathological details and treatment. The details of scrutiny and article selection are shown in Figure 1 [8].

Results

Demographics

A total of 13 case reports were included in this study. The major countries that contributed to this review included the USA, Korea, and Turkey (Table 1). Female patients were the majority, numbering 10 (76.92%), while male patients were three (23.08%). The mean age of the patients was 53.8 ± 12.59 years.

**Table 1:** Summary of case report included in the systematic review on gallbladder paraganglioma.

Study by	Title	Year of publication/country	Study type	Age/gender	Treatment
Ece et al. [9]	Gall bladder paraganglioma	2015/Turkey	Case report	57 y/F	Laparoscopic cholecystectomy
Mehra et al. [10]	Gall bladder paraganglioma	2005/USA	Case report	36 y/M	Cholecystectomy
Song et al. [11]	Gall bladder paraganglioma with hemorrhage: a case report and literature review	2021/Korea	Case report	48 y/F	Laparoscopic cholecystectomy
D’John et al. [12]	Primary gall bladder paraganglioma: a case report and review of literature	2020/USA	Case report	63 y/F	Laparoscopic cholecystectomy
Abdul Sater et al. [13]	Gall bladder paraganglioma associated with SDHD: a potential pitfall on F18-FDOPA PET imaging	2019/USA	Case report	36 y/F	Laparoscopic cholecystectomy
Baker et al. [14]	Mesenteric paraganglioma with gall bladder paraganglion nest	2012/UK	Case report	64 y/F	Laparoscopic cholecystectomy
Koplay et al. [15]	Gall bladder paragangliomas: computed tomography and magnetic resonance imaging findings	2014/Turkey	Case report	57 y/F	Laparoscopic cholecystectomy
Do et al. [16]	Gallbladder paraganglioma: a mysterious histological finding	2021/USA	Case report	53 y/M	Laparoscopic cholecystectomy
Cho et al. [17]	A case of hemorrhagic gallbladder paraganglioma causing acute cholecystitis	2001/Korea	Case report	45 y/F	Exploratory laparotomy
Aaquist et al. [18]	Synchronous detection of SDHA-related gallbladder paraganglioma and pancreatic neuroendocrine tumor	2020/Denmark	Case report	74 y/F	None
Yoshikawa et al. [19]	A case of lipid rich neuroendocrine tumor of the gall bladder mimicking a cholesterol polyp	2020/Japan	Case report	41 y/M	Laparoscopic cholecystectomy
Shreya et al. [20]	Case of the very rare gallbladder paraganglioma	2021/India	Case report	72 y/F	None
Xia et al. [21]	Case report: a rare case of primary paraganglioma of the gallbladder with a literature review	2023/China	Case report	48 y/F	Oral tyrosine kinase inhibitor a (Surufatinib)

Clinical Presentation

The commonest presenting complaint was right hypochondrium pain in seven (53.9%) cases, mimicking cholecystitis, while three (23.08%) patients were asymptomatic. Among those asymptomatic, two (15.38%) were admitted either for a surgical procedure (Roux-en-Y Gastric Bypass or Hepatectomy) or came in for an annual health check-up; one (7.69%) case. Otological symptoms like tinnitus, ear fullness, and watery discharge were present in two (15.38%) patients (Table 2). Among 13 patients, three (23.07%) had been diagnosed with paragangliomas of the head, neck, and mesenteries. 

**Table 2:** Most common presenting symptoms in patients of paraganglioma of the gallbladder, including frequency and percentage.

Presenting complaint	Frequency (n)	Percentage occurrence (%)
Right hypochondrium pain	7	53.85%
Asymptomatic	3	23.08%
Otological symptoms (tinnitus, ear fullness, watery discharge)	2	15.38%
Cough	1	7.69%

Past Medical History

About two (15.38%) patients had significant past medical history for hypertension and hypothyroidism, and carcinoma of the prostate (7.69%). About two (15.38%) cases had a significant family history of multiple endocrine neoplasia (MEN), ovarian cancer, and brain tumour.

Radiological Investigations

In seven (53.84%) reported cases, ultrasound abdomen was the first-line imaging modality, which revealed a mass in six (46.15%), most of which were incidental findings. The mean size of the mass was 1.74 ± 0.90 cm. This was followed by CT abdomen in six (46.15%). MRI and positron emission tomography/computed tomography (PET/CT) were also used in four (30.77%) and two (15.38%) cases, respectively. In PET/CT, 68Ga-DOTATATE (Gallium-68) yielded the most useful information (Table 3).

**Table 3:** Imaging modalities performed in reported cases, including frequency and percentage occurrence.

Investigations	Frequency (n)	Percentage occurrence (%)
Ultrasound (USG) abdomen	7	53.84%
Computed tomography (CT) abdomen	6	46.15%
Magnetic resonance imaging (MRI) abdomen	4	30.77%
Positron emission tomography/computed tomography (PET/CT)	2	15.38%

The average size of the lesion was 1.74 ± 0.90 cm. Fundus in five (34.86%) cases was found to be the most common location, followed by the three (23.08%) cases in the neck. Paragangliomas were found in the intramural layer in three (23.08%) cases, and intraluminal in two (15.38%), appearing as a polypoid or a lobular mass in the gallbladder.

Biochemical and Histopathological Features

Among all baseline investigations done in the patients, two (15.38%) had low haemoglobin (Hb), one (7.69%) showed elevated alkaline phosphatase (ALP), while three (23.08%) had normal liver function tests (LFTs) and carcinoembryonic antigen (CEA) levels. Neuron-specific enolase (NSE) and plasma dopamine levels were elevated in one (7.69%) case each.

There was a nested presence of chief and sustentacular cells in five (38.46%) cases, with an almost equal number of cases demonstrating the Zellballen pattern. A fibrovascular stroma was also visualised in three (23.07%) cases. Synaptophysin was the predominant chief cell marker; 11 (84.62%), and S-100 was the dominant sustentacular cell marker; 11 (84.62%) (Tables 4, 5).

**Table 4:** Histopathological features of the gallbladder, including frequency and percentage.

Histopathological features	Frequency (n)	Percentage occurrence (%)
Nested presence of chief cells and sustentacular cells	5	38.46%
Zellballen pattern	5	38.46%
Fibrovascular stroma	3	23.07%

**Table 5:** Immunohistochemical markers reported in the diagnosis of gallbladder paraganglioma, including frequency and percentage distribution.

Immunohistochemical markers	Frequency (n)	Percentage occurrence (%)
Chief cells markers		
Synaptophysin	11	84.62%
Chromogranin A	9	69.23%
CD56	3	23.08%
Vimentin	2	15.38%
Neuron-specific enolase	2	15.38%
Sustentacular cells marker		
S100	11	84.62%

Treatment

Laparoscopic cholecystectomy was done on almost ten (76.92%) patients, while open cholecystectomy was performed in another two (15.38%). An exploratory laparotomy was performed in only one patient (Table 6).

**Table 6:** Surgical interventions undertaken in reported cases, including frequency and percentage.

Surgical procedures	Frequency (n)	Percentage occurrence (%)
Laparoscopic cholecystectomy	10	76.92%
Open cholecystectomy	2	15.38%
Exploratory laparotomy	1	7.69%

The average duration of follow-up was 66.5 ± 82.10 weeks. Among the five (38.46%), no patient demonstrated signs of recurrence during the follow-up period, which was mentioned only for five patients. Follow-up information was not reported for eight (61.53%) patients. 

Discussion

Literature exists on the treatment protocols for pheochromocytoma and paragangliomas in locations such as the colon and pancreas. However, we discussed the management approach for paragangliomas arising specifically in the region of the gallbladder.

Primary GPG arises from the network of nerves innervating the gallbladder, namely, the hepatic plexus [20]. PGLs may produce hormones, such as adrenaline, norepinephrine, and dopamine. This depends on several factors, including their location (whether adrenal or extra-adrenal), certain genetic mutations, and their sympathetic or parasympathetic nature.

In our systematic review, GPG were diagnosed at 53.8 ± 12.59 years, and the majority of the patient population was female, coinciding with the literature, which also showed a higher preponderance in females [11].

Clinical Presentation

The clinical presentation of paragangliomas depends on whether they produce catecholamines or not. In the latter case, these exert a mass effect that is responsible for their symptomology [14]. In 12 (92.3%) cases reported in this review, the GPGs were non-functional, with suspicion of functionality in only one (7.69%) case report. The predominant symptom was right hypochondrial pain, seven (53.85%), presenting as cholecystitis, three (23.07%) were asymptomatic, and two (15.38%) had tinnitus and a watery ear discharge. None of the patients exhibited features specific to functional paraganglioma. Consequently, most lesions were discovered incidentally, with diagnostic investigations being directed primarily towards suspected cholecystitis.

The low occurrence of PGLs makes their diagnosis and treatment challenging [1]. Thus, to establish a diagnosis of PGLs, several factors, such as clinical features, biochemical and hormonal evidence of increased catecholamine production, imaging characteristics, histopathology, and genetic screening, are utilised [1].

Biochemical and Radiological Investigations

In our study, baseline tests, including complete blood count (CBC), liver function tests (LFTs), and, in pre-existing PGLs, carcinoembryonic antigen (CEA), carbohydrate antigen 19-9 (CA 19-9), plasma dopamine, plasma catecholamines, metanephrine, and chromogranin levels, were performed. Low haemoglobin was seen in 15.38% of patients, and one had elevated plasma dopamine levels at 435 pg/ml (normal: 0-30 pg/ml) with pre-existing PGLs.

Specialised biochemical testing for suspected PGLs includes catecholamines, vanillylmandelic acid (VMA), and metanephrines (MNs) levels; however, literature suggests that urine catecholamines and VMA may yield false negatives, while plasma free and urinary MNs offer better precision [22]. In our study, specialised biochemical testing was done in only one patient, which yielded normal results. In the silent SDHB subtype, where false negative results can be observed. Additional investigations with chromogranin A levels, imaging studies and non-specific neuroendocrine secretory proteins must be evaluated [1].

Literature also highlights the incidental discovery of PGLs, as they are detected incidentally on performing imaging for other unrelated causes. Literature suggests that in all patients with paraganglioma, whole body CT or MRI, or radionucleotide imaging should be considered to rule out metastatic disease/multiplicity preceding to surgery [3]. On CT, PGLs may be homogeneous or heterogeneous, necrotic with some calcifications, solid or cystic; however, they typically appear as solid, hypervascular, well-circumscribed masses with sizes varying from 1 to 15 cm [1]. Smaller-sized tumours are often homogenous, but larger tumours exhibit central necrosis [1]. Although MRI is not considered a first line for imaging, it is an appropriate choice for children, pregnant women, and patients with metastasis [22]. On MRI, particularly on T2-weighted imaging, the high and low signal intensity regions of the tumour appear in a characteristic “salt and pepper” pattern and classical "light-bulb" sign [4,11].

Functional imaging should play a vital role in establishing the diagnosis of malignancy, metastasis, course of disease in low tumour burden, the response to treatment and eligibility for radiopharmaceutical therapy [23]. The PET/CT technology has proven to be superior to scintigraphy, with single-photon emission computed tomography (SPECT/CT) having a better spatial resolution and a greater sensitivity [1]. Radionuclide imaging selection is based on germline/somatic mutations and clinical features [3]. A recent meta-analysis shows that the detection of PGLs by 68 Gallium-labelled somatostatin receptor analogues (SSAs) positron emission tomography/computed tomography ((68Ga)Ga-DOTA-SSA PET/CT) has the highest sensitivity of 93%, while that of dihydroxy-(18F)fluorophenylalanine ((18F)FDOPA) PET/CT is about 80%. 18F fluorodeoxyglucose ((18F)FDG) PET/CT (74%) has the lowest sensitivity of 74% [3]. Also, Ga-68 DOTATATE has significantly greater lesion-background contrast as compared to (18F) DG PET/CT. Other advantages of DOTATATE PET/CT include patient convenience, relatively low cost, and high specificity [24]. This aligns with our study, where 15.38% of patients underwent functional imaging, and Ga-68 DOTATATE was the most yielding.

Histopathological Features

On histopathology, 38.46% of specimens showed the nested presence of chief and sustentacular cells and the classic Zellballen pattern. On immunochemistry, 84.62% of chief cells were synaptophysin positive, and 72.72% were chromogranin A positive. About 84.62% of sustentacular cells were positive for S-100. This is similar to a study that suggested positivity for chromogranin A, synaptophysin, and S-100, pointing towards its neuroepithelial origin.

However, it is suggested that PGLs cannot be distinguished from other neuroendocrine tumours in instances where biomarkers are negative for keratin and site-specific transcription factors and positive for tyrosine hydroxylase (except for non-functioning head and neck PGLs). Here, GATA-3 immunohistochemistry can be used to confirm the diagnosis of PGLs [3].

Treatment

Treatment options include radiometabolic treatment, radiotherapy, chemotherapy, targeted therapy (i.e., antiangiogenic tyrosine kinase inhibitors), and peptide receptor radionuclide therapy (PRRT). For the elderly, frail, those having bilateral multicentric lesions or residual illnesses, may be candidates for watchful waiting or alternative nonsurgical treatment options such as radiotherapy [1].

In metastatic PGLs, the goal of treatment is to control tumour growth and survival, and to manage the manifestations of catecholamine excess [3]. Chemotherapy is preferred for progressive PGLs unsuitable for (131)I-metaiodobenzylguanidine (MIBG) or peptide receptor radionuclide therapy (PRRT) [1]. The most used regimen is cyclophosphamide, vincristine, and dacarbazine (CVD), considered the standard of care despite no prospective trials [3]. Additional therapies include tyrosine kinase inhibitors (TKIs), temozolomide (TMZ), other PRRTs such as Lu177-DOTATATE, and newer targeted therapies such as the HIF2a inhibitor belzutifan [23,25]. Although none of the cases in this review underwent chemotherapy, MIBG, or PRRT, these options are mentioned to reflect existing evidence on the management of metastatic paragangliomas.

The objective of surgery is to completely resect the tumour without rupture [1]. Surgical resection is the treatment of choice for localised abdominal and non-metastatic tumours [3]. In our study, the mainstays of treatment were laparoscopic cholecystectomy (76.29%), open cholecystectomy (15.38%), and exploratory laparotomy in 7.69% of patients. Literature favours open resection, though small, noninvasive, well-positioned tumours may be suitable for laparoscopic removal [22]. Preoperative catecholamine (CMN) alpha blockade is required for functional PGLs since hypertensive crises can be triggered in these cases due to CMN release [1,22].

Metanephrines and 3-methoxytyramine (plasma only)/CgA should be measured three to six weeks post-operatively. However, in non-functional PGLs and patients with postoperative elevation of metanephrines or 3-methoxytyramine, radionuclide imaging is recommended three to four months after surgery [1].

Paragangliomas are associated with the highest rate of germline susceptibility in cancer genetics. A hereditary form of paraganglioma should always be ruled out as nearly half of the cases are manifested as a syndromic presentation due to the existence of susceptible genes. Among these genes are those encoding for neurofibromin 1 (NF1), RET, VHL, menin (MEN1), SDH complex (SDHx: SDHA, SDHB, SDHC, SDHD), SDH complex assembly factor 2 (SDHAF2), TMEM127, MAX, FH, hypoxia-inducible factor 2A (EPAS1/HIF2A), EGLN1/PHD2, SLC25A11, and DLST [1]. The salient features that point towards the possibility of hereditary paraganglioma include family history, an early age of onset, and multiple primary neoplasms. In our study, 7.69% patients had a family history of multiple endocrine neoplasia (MEN). However, Mehra et al. did not specify the type of MEN, familial paraganglioma most commonly occurs in association with MEN type 2 [10].

In our study, follow-up was done in 38.46% of patients with no signs or symptoms of recurrence. Urinary fractionated MNs with or without plasma-free MNs, as well as CgA, are included as follow-up tests [1]. To rule out any residual disease, metanephrine levels should be measured two to four weeks after surgery. In biochemically quiet PCCs/PGLs, follow-up imaging is recommended every year or two [3].

Limitations

This review is limited by the rarity of gallbladder paragangliomas, with the analysis derived from only 13 case reports. Such limited data reduces the generalisability of the findings and restricts the strength of evidence-based recommendations. In addition, nearly all gallbladder paragangliomas described in this review were non-functional; however, most of the comparative literature is derived from functional paragangliomas. This disparity highlights a significant gap in the literature, making it difficult to extrapolate management strategies or prognostic outcomes for non-functional tumours.

Clinical Recommendations

**Figure 2:**
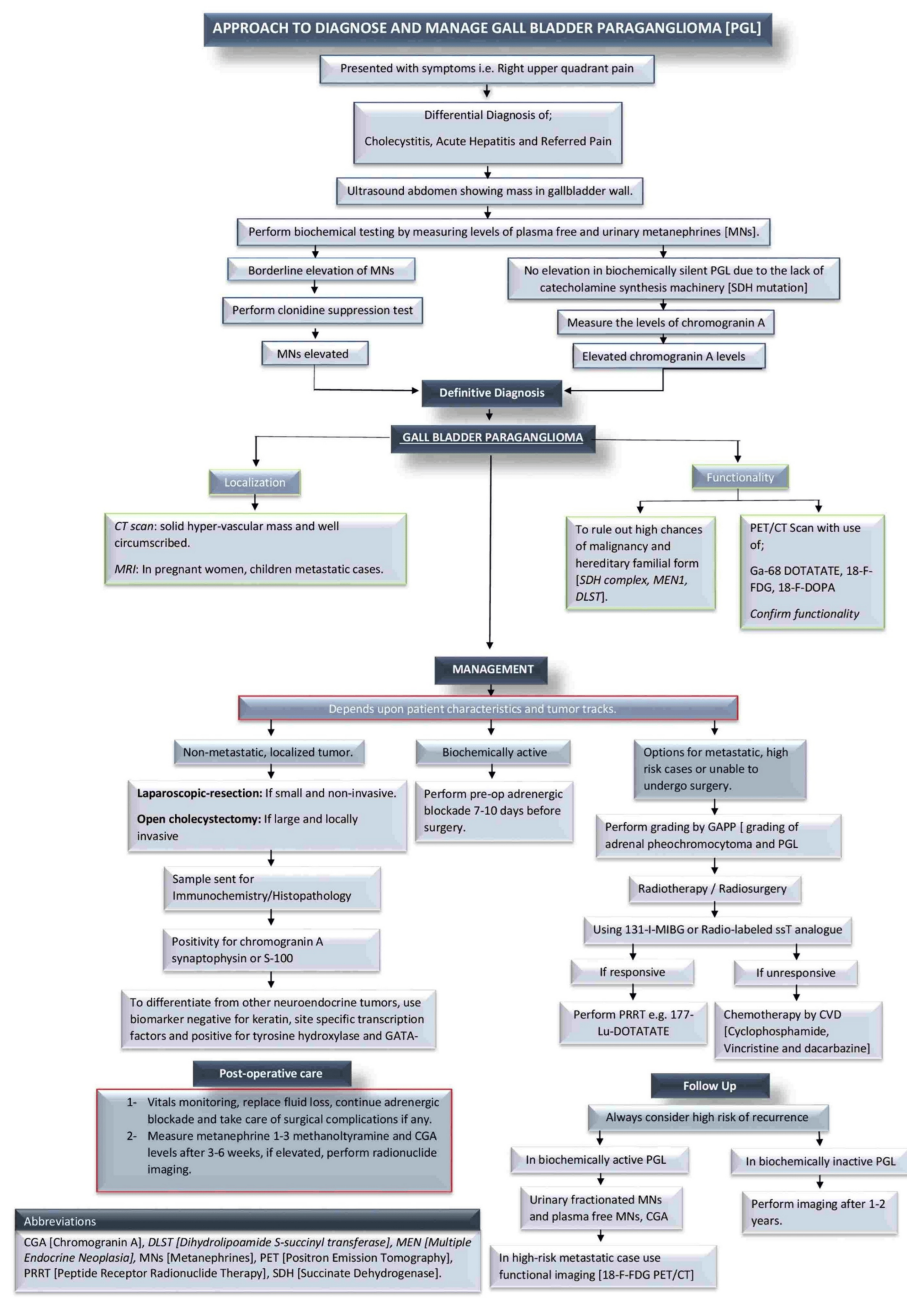
Author-proposed approach to diagnose and manage gallbladder paraganglioma (GPG). MN: metanephrine; PGL: paragangliomas; PET/CT: positron emission tomography/computed tomography.

Clinicians should keep gallbladder paraganglioma in the differential diagnosis of gallbladder masses, especially when patients present with atypical symptoms. In patients presenting symptomatically with a positive family history of neuroendocrine tumours, advanced imaging (CT, MRI, and functional imaging like MIBG or DOPA-PET) should be considered. Moreover, non-functional GPG can be proceeded directly to surgical resection following diagnostic confirmation. Long-term follow-up with surveillance imaging is recommended to detect recurrence or metastatic disease. After a thorough literature review of the included studies [9-21], the authors propose the approach to diagnosing and managing gall bladder paraganglioma (Figure 2).

## Conclusions

Gallbladder paraganglioma (GPGs) typically present with non-specific symptoms, often mimicking cholecystitis. In this review, all cases of GPG are mostly non-functional. They usually appear as a mass, with definitive diagnosis reliant on histopathological examination. However, the functional tumours are dealt with by approaches that exclude metastasis, association with genetic syndrome, and involve pre-operative alpha blockade. Surgical excision remains the most effective therapeutic option. However, the scarcity of cases and limited follow-up data restrict the ability to draw firm conclusions regarding recurrence risk and long-term prognosis.

## Appendices

Appendix A 

**Table 7:** The quality assessment of included case reports as per Joanna Briggs Institute critical appraisal.

Author	Q1	Q2	Q3	Q4	Q5	Q6	Q7	Q8	Overall appraisal
Ece et al. [9]	Y	N	Y	Y	Y	Y	Y	Y	Included
Mehra et al. [10]	Y	Y	Y	Y	Y	Y	N	Y	Included
Song et al. [11]	Y	Y	Y	Y	Y	Y	Y	Y	Included
D’Jhon et al. [12]	Y	Y	Y	Y	Y	Y	Y	Y	Included
Sater et al. [13]	Y	Y	Y	Y	Y	Y	N	Y	Included
Baker et al. [14]	Y	Y	Y	Y	Y	Y	Y	Y	Included
Koplay et al. [15]	Y	Y	Y	Y	Y	Y	Y	Y	Included
Do et al. [16]	Y	N	Y	Y	Y	Y	N	Y	Included
Cho et al. [17]	Y	Y	Y	Y	Y	Y	N	Y	Included
Aaquist et al. [18]	Y	N	Y	Y	Y	Y	N	Y	Included
Yosikawa et al. [19]	Y	Y	Y	Y	Y	Y	Y	Y	Included
Shreya et al. [20]	Y	Y	Y	Y	N	Y	N	Y	Included
Xia et al. [21]	Y	Y	Y	Y	Y	Y	N	Y	Included
